# Lessons From the Design and Rollout of an Electronic Medical Record System for Cervical Cancer Screening in Rwanda

**DOI:** 10.9745/GHSP-D-23-00469

**Published:** 2024-06-27

**Authors:** Nang’andu Chizyuka, Emily Crawford, Katharine Schilling Hebert, Sylvie Gaju, Inga Mumukunde, Jean Marie Vianney Dusengimana, Marc Hagenimana

**Affiliations:** a Clinton Health Access Initiative, Kigali, Rwanda.; b Clinton Health Access Initiative, Abuja, Nigeria.; c Clinton Health Access Initiative, Boston, MA, USA.; d Partners in Health, Kigali, Rwanda.; e Rwanda Biomedical Center, Kigali, Rwanda.

## Abstract

**Background:**

In its commitment to delivering comprehensive women's cancer early detection services, the Rwanda Ministry of Health rolled out a new cervical cancer screening program. The paper-based medical record system that tracked clients at different points in the continuum of cervical cancer care had challenges with storing data, accessing client information for follow-up visits, and fragmenting information on individual clients. To support the rollout of the new cervical cancer screening program, a new electronic medical record system was designed and implemented to ensure clients were followed along the complete continuum of care. We document the development and implementation of the electronic medical record system and highlight challenges and lessons learned during implementation.

**Methods:**

Implementation took a participatory approach to ensure that the electronic medical record system was efficient in tracking clients along the continuum of care. At every stage, a wide range of stakeholders were engaged, including clinicians, program managers, and software developers. Health facility visits and conversations were conducted with health care providers and data managers to review the existing system and ensure that the design and development of the electronic record system were suitable for the context in which it would be used.

**Results:**

Cervical cancer screening sites are currently using the electronic medical record system to document client information and track women along the continuum of care to reduce loss to follow-up. The system has been rolled out to all newly activated screening sites as part of national scale-up.

**Conclusion:**

Planning, collaboration, and adaptability were the key factors in this system’s successful rollout and should be the foundation of future data systems development.

## Abstract (precis)

We describe the process of designing, building, and implementing a cervical cancer electronic medical record system in Rwanda, highlighting experiences and lessons learned throughout this process.

## Teaser

**Key Messages:**

- The paper-based record system for monitoring cervical cancer care in Rwanda presented limitations with storing and accessing client information for follow-up visits and clinical decision-making along the continuum of care.
- To improve data quality, client management, and reporting in the context of a cervical cancer screening and treatment program, an electronic medical record system was developed and implemented in public health facilities in 16 of 30 districts of Rwanda.
- The successful development, rollout, and scale-up of the new system involved engaging stakeholders, incorporating end user feedback, and using adaptive training.

**Key Implication:**

- Program implementers, policymakers, and donors can gain valuable insights into effective system design, implementation, and sustainability, ultimately enhancing cervical cancer care and potentially serving as a model for similar endeavors in low-resource settings.

## INTRODUCTION

Health information systems are a critical component of health systems and, when functioning well, enhance tracking of progress, improve decision-making, and support the overall health system.^1^ Electronic medical record systems can improve quality of care, strengthen the relationship between clients and health care providers, and enhance compliance for follow-up treatment.^2^^–^^4^ The Government of Rwanda is committed to using digital health platforms to improve health care delivery to its population across various platforms. Over the past decade, the ICT Sector Strategic Plan of 2018–2024^5^ has informed the development and implementation of multiple digital health information systems, including electronic medical records. The Rwanda Ministry of Health developed a health information exchange system to enable the point-of-care systems currently being implemented in Rwanda to interoperate more easily with an integrated electronic medical record system. As part of the process, necessary infrastructure and equipment (e.g., computers and tablets) have been provided to facilities. The project is part of a nationwide digitization strategy that includes health information systems.

Cervical cancer is a global issue of public health importance that disproportionately affects women in low- and middle-income countries.^6^ Rwanda is considered a high-burden country for cervical cancer, with an annual incidence of 31.9/100,000 women and a mortality rate of 24.1/100,000.^7^ Rwanda has committed to addressing cervical cancer as a public health problem by strengthening and expanding screening and treatment of precancerous lesions. In the cervical cancer continuum of care, clients may contact the health system at multiple health facilities and time points. This dynamic continuum is difficult to track using paper-based systems, which often entail challenges with data quality, completeness, and timeliness. The paper-based system presented challenges of storage, difficulties in accessing client information for follow-up visits, and limited information for clinical decisions arising from fragmented information on individual clients. As part of the health system digitization strategy being undertaken in the country, the Government of Rwanda, together with partners, developed an electronic medical record system tailored for the revised national cervical cancer screening guidelines to improve data quality, storage, management, and use of client information, as well as ensure client follow-up along the continuum of care and increase reporting in the national health management information system (HMIS). This article shares the processes and lessons in the development and implementation of the cervical cancer electronic medical record system for cervical cancer screening.

The dynamic continuum of cervical cancer care is difficult to monitor with paper-based records systems.

## DEVELOPING AN ELECTRONIC MEDICAL RECORD SYSTEM FOR CERVICAL CANCER SCREENING

### Creating a Working Group for Development and Rollout

**FIGURE 1:**
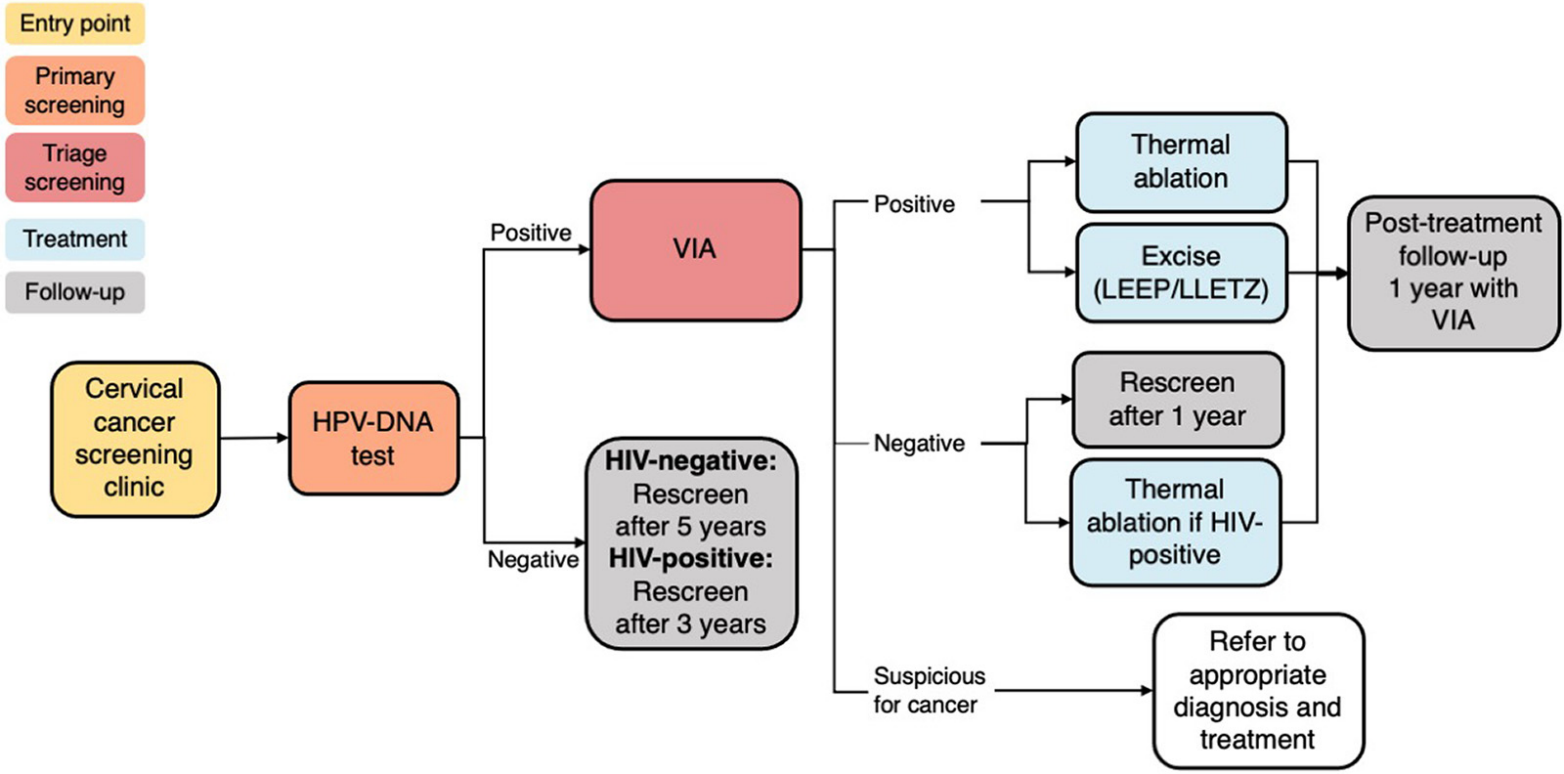
HPV-DNA Based Cervical Cancer Screening Algorithm, Rwanda^a^ Abbreviations: HPV, human papillomavirus; LEEP, loop electrosurgical excision procedure; LLETZ, large loop excision of the transformation zone; VIA, visual inspection with acetic acid. ^a^Based on World Health Organization cervical cancer screening guidelines.^7^

A working group was created to support and manage the design, development, and implementation of the electronic medical record system. This working group comprised software developers, health care providers from public health facilities, and project staff from Rwanda Biomedical Center (RBC), Partners in Health, and Clinton Health Access Initiative (CHAI) implementing a human papillomavirus (HPV) DNA test-based cervical cancer screening and treatment program. Special attention was paid to involving health care providers in the design process as they would be the main users of the system and would provide insight into how clinical workflows could be redefined in the new system and help select the final system. Similar projects have attributed their success to engaging stakeholders in pre-implementation design, nurturing local champions, and encouraging local leadership.^2^ This leadership was particularly important for this project, as the electronic system was intended to coincide with the rollout of the Government of Rwanda’s 2020 revised cervical cancer screening guidelines that included the HPV DNA test-based screening (Figure 1).^7^ The new guidelines informed the implementation model for the national scale-up that commenced in 2021. Screening services are both opportunistic and population based in both routine services and campaigns.

### Identifying Data Requirements

First, the working group reviewed the existing paper-based screening form that collected clients’ information based on the existing clinical guidelines and compared the form to the revised screening algorithm (Figure 1) to identify areas that needed to be modified.

**FIGURE 2:**
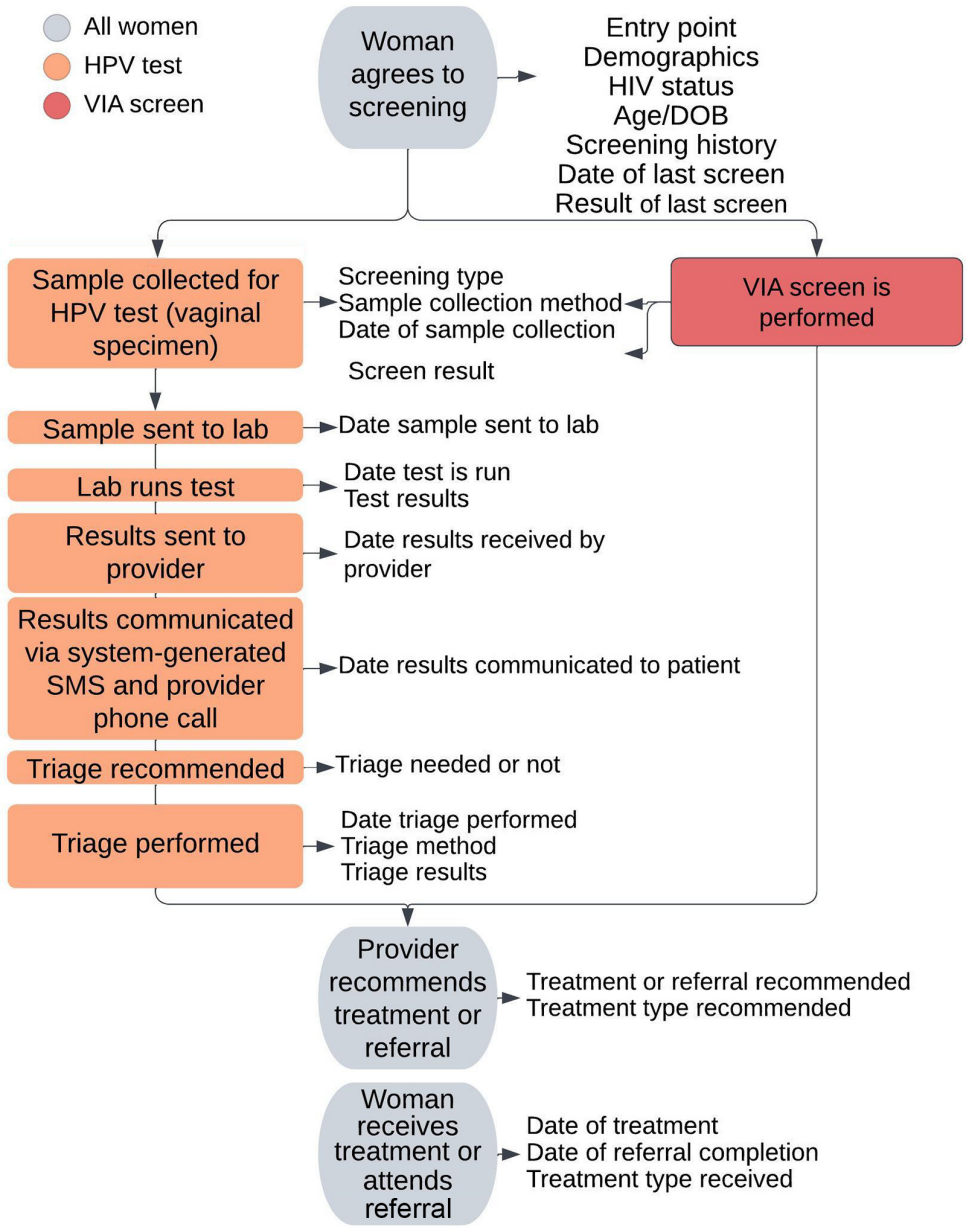
Data Points Required at Each Clinical Stage of the Cervical Cancer Screening and Precancer Treatment Process Abbreviations: DOB, date of birth; HPV, human papillomavirus; SMS, short message service; VIA, visual inspection with acetic acid.

This assessment process involved health facility visits and conversations with health care providers and data managers, the end users, to understand the context where the system would operate. From these conversations, the working group compiled a list of all data that needed to be collected (Figure 2) at each clinical stage of the cervical cancer screening and treatment process alongside the required flow of information.

The data and clinical workflows (Figure 2) were presented to the software developers for their review and input and were shared widely among stakeholders in the cervical cancer space for validation. In addition, the working group conducted a desk review of existing cervical cancer guidelines to ensure no relevant data points had been missed. This formed the basis for selecting a suitable software architecture to develop the electronic medical record system.

### Identifying Software Requirements

The working group identified several requirements for the electronic medical record system. In addition to the capacity to collect all the data points (Figure 2) identified in the cervical cancer screening process, the primary requirement was a system that could send an automated short message service to individuals to reduce loss to follow-up. The cervical cancer module on OpenMRS was deployed to support screening services that were being expanded to the health center level in Rwanda, where the Internet was unreliable, so it was also important for the system to allow offline data collection and referral of clients to higher-level facilities.

The working group identified that the new electronic medical record system had to enable data collection at all points in the continuum of care, send automated reminders to clients for follow-up, enable offline use, and use existing systems to save money and time.

Finally, the working group preferred to work with a software system that was already in place in Rwanda for cost efficiency, time savings, and user familiarity. Rwanda currently uses 3 electronic medical record systems for individual-level client information: DHIS2 for reporting at all levels and Open Clinic and OpenMRS, which are used at all hospitals for electronic medical records.

### Defining the System’s Objectives

Then, the working group identified and defined the objectives of the electronic medical record system based on information derived from observing the pitfalls of the paper-based system and prior experiences using similar electronic systems. The objectives of the proposed system were to allow systematic, secure storage of client information; enable easy provider access to client records; improve the use of data in providing support to clients; integrate data to the national HMIS; and improve the quality of data through accuracy and completion.

### Selecting the System

Of the 3 electronic medical record systems already in use in Rwanda at the point of care, OpenMRS and DHIS2 met the proposed system objectives, particularly the need for offline data collection. Guided by the defined objectives, the working group selected OpenMRS to develop and implement the cervical cancer screening electronic medical record system.

OpenMRS, an open-source electronic medical record system, captures individual client information in a standardized format that is then automatically aggregated and linked to the national HMIS for reporting. OpenMRS uses a data dictionary concept that makes it easy to customize for different disease programs without having to reprogram, making it ideal for low-resource settings that usually have a challenge of technical expertise to support complex programming tasks.^8^ In Rwanda, OpenMRS was first used in 2006 for a Partners in Health-supported HIV program, where it was used to support initiating therapy and monitoring treatment.^9^ In 2020, the cervical cancer electronic medical record system was developed as a module on an existing OpenMRS by the Rwanda Ministry of Health and RBC with support from CHAI and Partners in Health.

### Resourcing the System’s Development

After deciding to use OpenMRS, the working group developed a work plan detailing project timelines and requirements. The most critical requirement for the project’s success was to ensure the identification of dedicated software developers. The technical working group identified software development capacities within the working group and external resources that could be engaged when needed. Other key requirements were Internet bundles and hardware components, such as tablets and processors. Implementing partners made commitments to procure the hardware components required for the project.

## IMPLEMENTING OPENMRS FOR CERVICAL CANCER SCREENING

### Training Users on OpenMRS

After developing the electronic medical record system, the project team developed a plan to train health care providers and data managers on the necessary technical skills to operate the new electronic medical record system. The software developers trained selected health care providers as clinical master trainers using a training-of-trainers approach. These master trainers then trained other health care providers and data managers at the district level. The training approach focused on hands-on experience with OpenMRS.

Training on the new clinical guidelines was rolled out alongside the training for OpenMRS. However, it quickly became evident that this approach did not allow sufficient time for trainees to become comfortable with the electronic medical record system. To resolve this issue, the training plan was updated, and district information technology officers were subsequently trained as both master trainers and district technical support focal persons. The training equipped these district information technology officers with the skills necessary to conduct system administrative tasks and troubleshooting and simultaneously offered an expanded pool of trainers for the program rollout.

### Collecting End User Feedback

**FIGURE 3:**
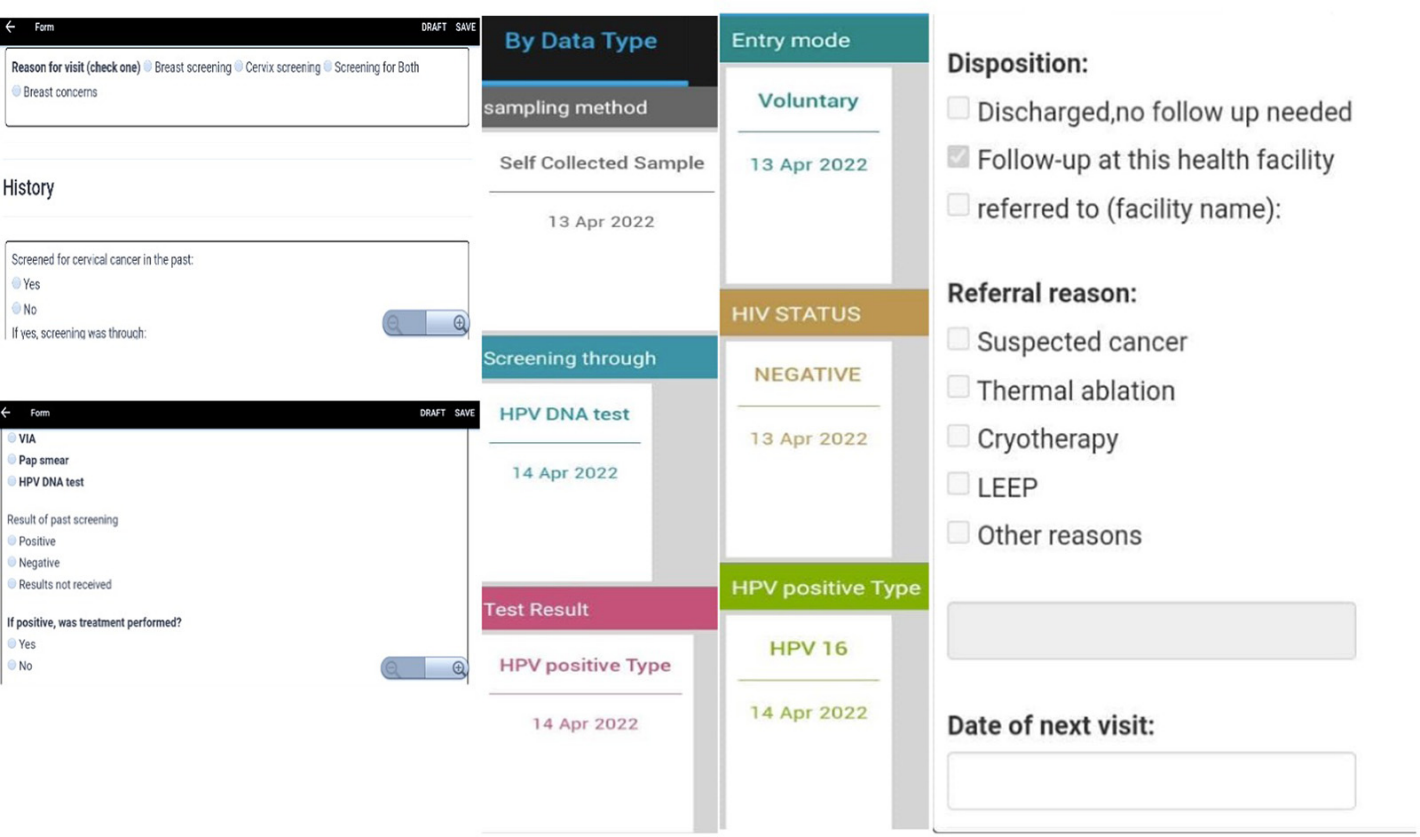
Screenshots of the Screening Form and Information Outputs in the Electronic Medical Records System Abbreviations: HPV, human papillomavirus; LEEP, loop electrosurgical excision procedure; VIA, visual inspection with acetic acid.

Cervical cancer screening services were introduced at the health center level in 2020 at the same time as the rollout of the electronic medical record system. The rollout was phased to allow feedback and improvement of the system before scale-up. After deployment in 3 districts, the working group convened to consolidate feedback from end users, program managers, and system administrators for further improvement of the system. Health care provider feedback centered around the appearance of the screening form on the tablet and the absence of skip patterns that required more time to read. Users provided suggestions on additional features, such as having client lists for results, referrals, and missed visits to support their clinical processes. Based on this feedback, the screening form (Figure 3) was improved, and reports were designed to allow easier access to both clinical and programmatic data.

### System Scale-Up

**FIGURE 4:**
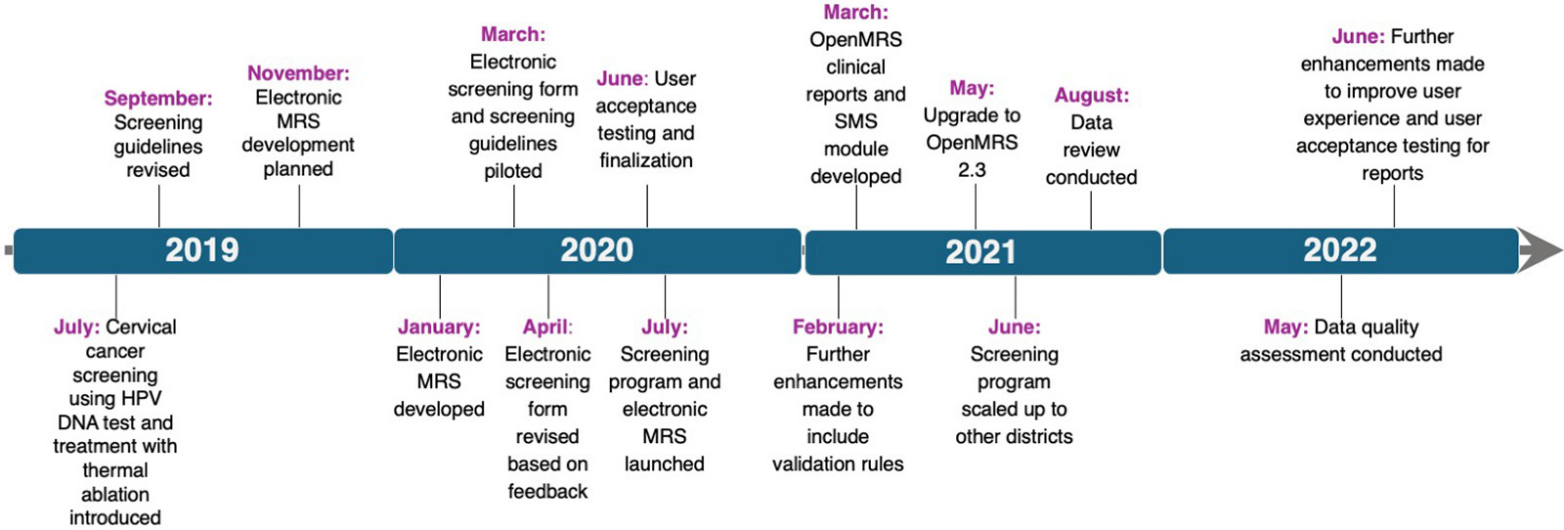
Implementation Timeline for the Rwanda Cervical Cancer Screening Program and Electronic Medical Records System Development Abbreviations: HPV, human papillomavirus; MRS, medical records system.

After meeting all the requirements during user acceptance testing, the system was ready for scale-up in time for the launch of the screening program (Figure 4). At the end of 2022, the system had been deployed to all public health facilities in 16 of 30 districts in the country and more than 150,000 client records had been entered. The deployment plan for the electronic medical record system was to have it be used alongside a paper register for 6 months. The system replaced the paper system following data quality assessments with successful outcomes.

The cervical cancer electronic medical record system is expected to be deployed to all 30 districts in the country along with the national cervical cancer screening program scale-up by the end of 2025. Data from the system have been used to monitor the implementation of the national cervical cancer prevention program at facility, district, and national levels. Information on screening volumes, test results, treatment, and referrals can be accessed on the web platform in real time. The system is being used not only in health facilities but also in laboratories where results are entered into the system and made available to providers in real time. The use of the system has contributed to a reduction in clients lost to follow-up. For example, 90% of HPV-positive screened women in Gasabo district (Kigali) returned to the facility for triage and treatment with thermal ablation or loop electrosurgical excision and referrals in accordance with the screening algorithm. Data use is expected to increase when automated reports are launched, as this will make aggregated data and vital client lists available at all levels of the continuum of care in real time. Further system enhancement is underway to integrate it with laboratory systems for automated transmission of results as part of laboratory systems optimization and integration with the national HMIS for reporting. The HMIS reports have been developed pending integration into the DHIS2-based HMIS.

## LESSONS LEARNED

### Platform Design

**Establishing a dedicated working group with a wide range of stakeholders supported the project at every stage.** This working group consisted of a wide range of stakeholders, including clinicians, software developers, and programmatic personnel, and deliberately incorporated members who had prior experience with similar projects in Rwanda using OpenMRS and DHIS2. This not only provided expertise but also helped to ensure sustainability, as in many resource-constrained countries, projects are challenged by limited expertise to develop, install, and maintain systems.^10^

**Incorporating clinical realities and feedback from target end users allowed the system to be more realistic and contextualized.** The working group deliberately engaged target end users throughout the process by visiting health facilities, including health care providers in the working group, and collecting and incorporating feedback from users after pilot implementation. This ensured that the system met users’ needs and enabled the collection of all the data providers needed to collect. The use of participatory design early in the development process allowed for a more realistic and contextualized electronic medical record system.^11^

**Ensuring that everyone understands the technical concepts of the system helped better inform the system’s development**. Early in the project, the working group recognized that many programmatic team members did not understand the discussions with developers about technical requirements. To address this, the technical team conducted a workshop for all working group members to learn basic information on the functionality of OpenMRS and what was required of the program team to inform the development of the system. This workshop aligned stakeholder expectations and created a platform to discuss how the electronic medical record system would interact with other systems and processes, such as the laboratory and national HMIS.

### Testing and Implementation

Several important elements were critical to the successful rollout of the new electronic medical record system.

**Using an adaptive training approach ensured effective rollout**. Clinicians, data managers, and ultimately, district information technology officers were trained in the use and maintenance of the system. This not only mitigated a challenging rollout process, where both clinical guidelines and data collection were being revamped, but also allowed for a more decentralized technical support structure. For future projects, conducting a critical assessment of basic competencies required to lead capacity-building may support creating the most effective teams for rollout. In the cervical cancer program’s experience, the most effective approach was to have the clinicians train alongside the information technology officers to bring together the clinical flow and the navigation of the electronic medical record system. This process proved more effective in enhancing learning among health care providers and allowed information technology officers to integrate clinical information into the training approach.

**Integrating user feedback improved uptake**. During the initial pilot rollout, the electronic screening form was field tested among providers, which gave important information to the software developers to ensure the system workflow was harmonized with the clinical flow. This step was essential to success, as health care providers more readily accept electronic systems that provide clinically useful information.^12^ After this feedback collection, the OpenMRS system was updated, and a pilot rollout was conducted. During the pilot, the working group, including the developers, maintained regular communication with users for feedback on the system. In addition, the working group took advantage of the web-based operating system OpenMRS to monitor data quality in real time and engage users for validation. This kept the developers in close touch with the system functionality and other areas for development. This monitoring was also cost-effective, as initial data quality checks could be conducted remotely.

**Identifying dedicated resources for system maintenance ensured sustainable rollout**. The participatory nature of the program and the ongoing training of the working group members ensured that multiple roles were available to support the long-term implementation of the system. Engagement of district information technology officers for training and system maintenance also provided ongoing support. Finally, the RBC engaged a software developer to complete technical tasks that came up during testing and implementation. The external consultant was part of the review and design workshop, which provided a platform to share the details and specifications of the electronic system.

## CONCLUSION

Designing an electronic medical record system requires carefully assessing and understanding the context in which it will be implemented. Careful planning before implementing the system in Rwanda, coupled with the engagement of key stakeholders at every step, guided the implementation process and minimized the challenges encountered. Regular communication among stakeholders allowed for the mitigation of challenges and points of reflection to crystallize lessons learned. Engaging stakeholders with prior experience in similar projects also contributed greatly toward the project’s success.

Those who plan to implement similar projects should focus on harnessing existing knowledge and resources, ensuring collaboration and contribution from users at all levels, and planning for an agile implementation with an ability to shift strategy and adjust the final design even after rollout if needed. Planning, collaboration, and adaptability were the key factors in this system’s successful rollout and should be the foundation of future data systems development.

## Notes

**Peer Reviewed**

**First published online:** April 29, 2024.
